# Nocturnal Road Traffic Noise Exposure and Children’s Sleep Duration and Sleep Problems

**DOI:** 10.3390/ijerph14050491

**Published:** 2017-05-06

**Authors:** Kjell Vegard Weyde, Norun Hjertager Krog, Bente Oftedal, Jorunn Evandt, Per Magnus, Simon Øverland, Charlotte Clark, Stephen Stansfeld, Gunn Marit Aasvang

**Affiliations:** 1 Department of Air Pollution and Noise, Norwegian Institute of Public Health, Pb 4404 Nydalen, 0403 Oslo, Norway; NorunHjertager.Krog@fhi.no (N.H.K.); Bente.Oftedal@fhi.no (B.O.); Jorunn.Evandt@fhi.no (J.E.); 2 Domain of Health Data and Digitalization, Norwegian Institute of Public Health, Pb 4404 Nydalen, 0403 Oslo, Norway; Per.Magnus@fhi.no; 3 Institute of Health and Society, Faculty of Medicine, University of Oslo, Pb 1130 Blindern, 0318 Oslo, Norway; 4 Division of Mental Health, Norwegian Institute of Public Health, Zander Kaaes gate 7, 5015 Bergen, Norway; Simon.Overland@fhi.no; 5 Faculty of Psychology, University of Bergen, Pb 7807, 5020 Bergen, Norway; 6 Centre for Psychiatry, Wolfson Institute of Preventive Medicine, Barts and the London School of Medicine, Queen Mary University of London, Charterhouse Square, London EC1M 6BQ, UK; c.clark@qmul.ac.uk (C.C.); s.a.stansfeld@qmul.ac.uk (S.S.)

**Keywords:** road traffic noise, children, sleep duration, sleep problems, socioeconomic status

## Abstract

Almost half of the European Union (EU)’s population is exposed to road traffic noise above levels that constitute a health risk. Associations between road traffic noise and impaired sleep in adults have consistently been reported. Less is known about effects of noise on children’s sleep. The aim of this study was to examine the association between nocturnal road traffic noise exposure and children’s parental-reported sleep duration and sleep problems. The present cross-sectional study used data from The Norwegian Mother and Child Cohort Study. Parental report of children’s sleep duration and sleep problems at age 7 was linked to modelled levels of residential night-time road traffic noise. The study population included 2665 children from Oslo, Norway. No association was found between road traffic noise and sleep duration in the total study population (odds ratio (OR): 1.05, 95% confidence interval (CI): [0.94, 1.17]), but a statistically significant association was observed in girls (OR: 1.21, 95% CI: [1.04, 1.41]). For sleep problems, the associations were similar (OR: 1.36, 95% CI: [0.85, 2.16]) in girls. The ORs are presented for an increase of 10 dB. The findings suggest there is an association between road traffic noise and sleep for girls, underlining the importance of protecting children against excessive noise levels.

## 1. Introduction

Sleep disturbance is one of the main negative consequences of environmental noise [1]. About 40% of the European Union (EU)’s inhabitants, 125 million people, are exposed to traffic noise levels exceeding the value of L_den_ 55 dB [2], a level likely to be harmful to health [3]. More than 25 percent of Norwegian children are exposed to noise above L_den_ 55 dB, and this proportion is increasing [4]. In addition to impact on sleep, environmental noise is associated with annoyance, cognitive impairment, cardiovascular diseases, and mental health impairments [1,2,5,6,7]. 

Knowledge about the association between nocturnal road traffic noise and sleep mainly comes from studies on adults, and has been obtained with different study designs and different assessments of sleep and noise [8,9,10,11,12,13]. Few studies exist on noise and sleep in children [14]. The World Health Organization [1] has suggested that future epidemiological noise and health studies should focus on children and other vulnerable groups. Children go to bed earlier than adults, and road traffic noise levels are typically higher in the evening than at night [15]. Previous studies have found associations between environmental noise and negative outcomes in children, such as impaired cognition, reduced wellbeing, increased negative behavior, and increased annoyance and stress [6,16,17]. Sleep may be a mediating factor in these noise and health associations. Sleep promotes neuroplasticity, neurodevelopment, and neuronal function and is important for maintaining good health and optimal daily functioning [18,19,20]. Given the extensive brain development that takes place during childhood and the academic and lifestyle key factors that start developing early in life, sleep may be especially important for children [21]. Thus, for children who are newcomers in the school system, sufficient sleep is important to get a good start in school, both academically and socially. It is therefore of great concern that children’s sleep duration has been declining over the last several decades [22,23]. 

Previous studies on children have shown that road traffic noise is associated with difficulties falling asleep and presence of “any sleeping problem” [24], restless sleep, and difficulties falling asleep after nocturnal awakening [25]. Furthermore, an association with daytime sleepiness is reported [13]. An experimental study found decreased subjective sleep quality ratings when the noise level was increased [26]. In that same study, reduced polysomnography-measured sleep onset latency was found after reducing road traffic noise exposure. A couple of studies used actigraphs to assess sleep. One found no associations between noise and sleep [13]. The other found a slight tendency for children with shorter sleep duration to report that noise outside kept them from sleeping well [27]. 

To date, large studies on noise and sleep in children are lacking. No population based study so far has examined the impact of nocturnal noise on children as young as 7 years old. In addition, the potential moderating role of gender and socioeconomic status (SES) remain two almost unexplored topics. Gender differences in sleep among adults is consistently reported [28]. A recent Norwegian study of 16–18 year-olds found longer sleep onset latency and higher insomnia rates for girls, and later bedtimes for boys [29]. The few studies on noise and sleep in adults that have looked at gender differences have obtained mixed results [30,31,32]. Although not all studies on children’s sleep have found gender differences [22], girls are often found to sleep longer than boys [33]. Concerning SES, children from areas of lower SES are particularly vulnerable to sleep impairments [33]. Both lower income and lower maternal education level are associated with reduced time in bed [34]. These children also tend to be exposed to road traffic noise to a greater extent than children from high-SES families [4]. Road traffic noise, therefore, may not only have a negative impact on sleep and health, but also increase social health differences among children. 

The main aim of the present study was to examine the association between night-time road traffic noise and parental-reported sleep duration and sleep problems among children aged 7 years. We hypothesized that increased road traffic noise exposure would be associated with shorter sleep duration and increased probability for sleep problems. It was also explored whether this association differed between genders, and between children of different socioeconomic statuses.

## 2. Materials and Methods 

### 2.1. Study Population

The study used questionnaire data from the Norwegian Mother and Child Cohort Study, MoBa [35]. MoBa is a prospective population-based pregnancy cohort study conducted by the Norwegian Institute of Public Health. Participants were recruited from all over Norway from 1999–2008. The women consented to participation in 41% of the pregnancies. The cohort now includes 114,500 children, 95,200 mothers, and 75,200 fathers. Mothers received invitations by mail, along with appointments for ultrasound scanning in week 17 or 18 of pregnancy. No exclusion criteria were used in the main study. Three questionnaires were mailed to the mothers during pregnancy, and when the children were 6, 18, and 36 months, and 5, 7, and 8 years. The cohort is described in more detail elsewhere [35]. The present study is based on version 9 of the quality-assured data files.

**Figure 1:**
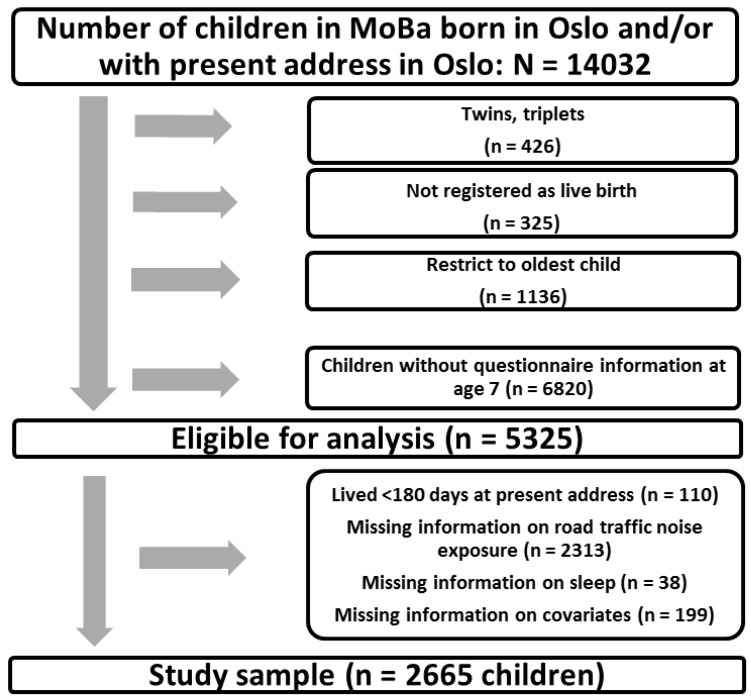
Flow chart showing the selection of the study sample.

From an initial sample of 14,032 MoBa participants who at some point had been registered with an Oslo address, we excluded the following: multiple births, births not registered as live birth, all but the oldest participating child of each mother (to avoid multiple dependent observations), and children lacking questionnaire information at age 7. Five thousand thee hundred twenty-five (5325) children met these criteria. Further, children having lived less than 180 days at the present address (and who therefore may not yet have “returned to normal” after the possible stressful life-changing event of moving to a new place), or who had missing values on either road traffic noise, sleep duration, or any covariates, were excluded. The final study sample contained 2665 children born between 2001 and 2008 (see Figure 1). Participants with addresses in Oslo were selected because of the availability of noise exposure estimations for this city. Children from all of the 15 urban districts of Oslo were present in the study. 

### 2.2. Noise Exposure Assessment 

Exposure to traffic noise was modelled at the child’s residential address at the time the 7-year questionnaire was completed. The A-weighted evening (19:00–23:00) and night-time (23:00–07:00) equivalent noise level, L_en_, based on annual average daily traffic (AADT) with diurnal distribution was employed. The L_en_ was used as the noise metric to cover the time children typically spend in bed, and was estimated for the most exposed façade of each child’s residence. Noise from road traffic was the main exposure variable, but residential exposure to noise from rail traffic (tram, railroad) was also modelled. The road traffic noise variable was continuous. Estimations of road and rail traffic noise exposure were conducted by the Agency for Urban Environment, the City of Oslo according to the Environmental Noise Directive [36]. The Nordic Prediction Method [37,38] and the software program CadnaA version 4.3 (DataKustik GmbH, Germany) [39] with a geographic information system (GIS), were used. The children’s residential addresses at the time of questionnaire completion were geocoded and grid predictions of 5 x 5 m^2^ at 4 m height were used to assign noise to the geocoded addresses. The noise assessment was based on input data for the year 2011 and included data on topography, building polygons, traffic counts (estimations for smaller roads without counts), estimated values for 24 h traffic distribution (75% day, 15% evening, and 10% night for highways, and 65%, 20%, and 15% for municipal roads), signed speed, information on noise barriers, and ground surface (hard or soft). The search radius of 1000 m was used for highways, and 500 m for municipal roads. Residential exposure to rail traffic noise was modeled separately and in a similar way as road traffic noise. For rail traffic, rail time tables were used to obtain information on traffic volume and diurnal distribution of traffic. The rail traffic noise variable was categorized as unexposed (residential address not within a radius of 700 m from a railway line and 300 m for trams and metros), exposed to 0–30 dB, or exposed to >30 dB (both exposed groups lived within a radius of 700 m from a railway line and 300 m for trams and metros). Outside these radii, the rail traffic noise is either nonexistent or is so low that it is masked by other noise sources. Seventy-four (74) children had their questionnaire filled in before year 2011. For these children, noise levels were compared to estimations based on 2006 data. If differences were >3 dB and changes likely to affect noise exposure (buildings road structure etc.) had occurred between 2006 and questionnaire completion date, the 2011 estimations were used. If changes had occurred between completion and 2011, values based on 2006 estimations were used. If no changes had occurred, values based on 2011 estimates were kept.

### 2.3. Sleep Outcomes

Sleep duration was used as the main outcome variable. In the MoBa 7-year-questionnaire, mothers were asked: “Approximately how many hours of sleep per night does your child usually obtain on weekdays?” with five different response categories: 8 h or less, 9 h, 10 h, 11 h, and 12 h or more. The variable was recoded into three categories to avoid small or empty cells: Sleeping less than 10 h, sleeping 10 h, or sleeping more than 10 h. Ten hours corresponds approximately to the mean sleep duration of children aged 7 years [22]. Information on sleep problems were also obtained from the MoBa 7-year-questionnaire. Mothers were asked whether their children ever have had sleep problems, and if so, when the problems started and finished. The sleep problem variable was scored 0 if no problems were reported, or if the problems were reported to have disappeared. If problems were reported and they still were present, the variable was scored as 1. 

### 2.4. Covariates

Covariate information was obtained from the MoBa 7-year questionnaire, the Medical Birth Registry of Norway, and Statistics Norway. Covariates were selected using Directed Acyclic Graphs (DAG; see Figure S1). DAG is a tool used for covariate selection to minimize the magnitude of bias [40,41,42]. The DAG was developed after an extensive literature review, documenting the covariates’ associations with exposure and outcome. Based on the DAG, a minimal adjustment set (the minimal selection of variables to be adjusted for in order to avoid a biased result) was suggested using dagitty.net [43]. This minimal adjustment set included gross household income, season when the questionnaire was completed, and urbanity. An additional set of variables was considered important to include in the full model because of their well-established association with exposure and outcome, and because they were well measured (few missing values, registry based, etc.): mother’s education, ethnicity, siblings in child’s household, and type of residential building. Gender and age (months) were also included. Table 1 gives a more detailed description of the covariates.

### 2.5. Statistical Analysis

To investigate the association between nocturnal road traffic noise and children’s sleep duration, ordered logistic regression was used. The proportional odds assumption, as tested with an approximate likelihood ratio test for ordinal models (omodel logit test) [44] and Brant test, was violated for variable ethnicity. In addition, the homoscedasticity assumption was tested with a stepwise selection procedure, and the same variable, ethnicity, was identified as a potential source of heteroscedasticity. A heteroscedastic ordered logistic regression model [45] was used to deal with the assumption violations. First, a crude model was fitted, including only road traffic noise, age, and gender. Second, a minimal adjustment set model was fitted, additionally including income, season, and urbanity. The full model included all covariates listed above. Interaction terms between road traffic noise and gender, road traffic noise and mother’s education (categorical, high school, max four years of university or college, or five or more years in university or college), and road traffic noise and household income (continuous, gross income in Norwegian kroner (NOK)) were included in the full model. Likelihood ratio tests were used to evaluate whether gender, income, and education were significant effect modifiers in the association between road traffic noise and parental-reported sleep duration. Based on those test results, models were stratified by gender. Two sensitivity analyses were done. One included only children who lived with both parents. Children with shared custody had an unknown residential traffic noise exposure for the time spent at their fathers’ residence, whereas we assumed no such noise exposure “gaps” for children not switching between parents’ addresses on a regular basis. In the other sensitivity analysis, rail traffic noise was included as a covariate, since studies on adults have found associations between rail traffic noise and sleep [46]. Thus, for each gender, five different models were analyzed: (1) a crude model with nocturnal road traffic noise and age as independent variables; (2) a minimal adjustment set model also including income, season, and urbanity; (3) a full model with all covariates listed above; (4) and (5) models testing the interaction between road traffic noise and income and education. The sensitivity analyses were done with model 2. A 5% significance level was used in all models, except for the tests of interaction terms, where the level was set to 10%. A 10% level has also been used in other studies [9], and we decided to use it as well, since, generally, very large studies are often needed to find statistically significant interactions using a 5% level. Odds ratios (ORs) are reported per 10 dB increase in road traffic noise. Smooth functions were estimated for boys and girls separately, using non-parametric regression with cubic spline as smoother (Generalized Additive Models, GAM). A full logistic regression model using penalized likelihood was used to assess the association between road traffic noise and sleep problems. In addition, this model was run with interaction terms between road traffic noise and gender, and stratified by gender. Chi-square analyses and ANOVAs were performed to examine whether the 7-year-olds in the study sample (n = 2665) differed on important covariates from the other MoBa children from the initial Oslo population who were not part of the study population (n = 8582). The covariates compared included gender, ethnicity, urbanity, household income, maternal education, siblings under 4 years of age, type of residence, and road traffic noise. The non-sample group was not part of the study sample either because they had dropped out from the MoBa study, moved out of Oslo before age 7, had not yet reached the age of 7, and were thus still waiting to answer the 7-year-questionnaire, or had missing values on any of the included variables. Information on the covariates were obtained from Statistics Norway, the Medical Birth Registry of Norway, and Health and Welfare Agency, City of Oslo, and was available for all the children, regardless of whether the 7-year questionnaire was completed or not. Similar comparisons were done between genders. 

Analyses were done in Stata version 14.0 (StataCorp, College Station, TX, USA) [47]. Splines were created in R version 3.2.2 (R Core Team, Vienna, Austria).

The present study was approved by the Regional Committee for Medical and Health Research Ethics in Norway (project: 2014/205/REK sør-øst B; date of approval: 25 March 2014). Investigations were carried out according to the Declaration of Helsinki. The establishment and data collection in MoBa has obtained a license from the Norwegian Data Inspectorate and approval from The Regional Committee for Medical Research Ethics. All subjects gave their informed consent for inclusion before they participated in MoBa.

## 3. Results

### 3.1. Descriptives

Compared to girls, a higher proportion of boys slept less than 10 h. A slightly higher proportion of girls slept more than 10 h. More than half of both the girls (58%) and the boys (57%) slept approximately 10 h. There was a tendency for children with low-educated mothers to have shorter sleep duration (estimates not shown). Household income was not associated with sleep (estimates not shown). The mean road traffic noise level for the total population was L_en_ 47.2 dB (standard deviation [SD] = 7.8). For girls, it was L_en_ 47.5 dB (SD = 7.7), and sleep duration tended to increase as the average noise level decreased. For boys, the average noise level was L_en_ 47.0 dB (SD = 7.9), and it remained approximately the same across the sleep duration categories. The L_en_ indicator for road traffic noise correlated highly with L_n_ (Pearson’s r = 0.99; L_n_ mean: 45.2; L_en_ mean: 47.2), which is the indicator used in the Night Noise Guidelines [48]. A total of 52.3 percent of the children (n = 1393) were exposed to rail traffic noise, with a mean of L_en_ 33.8 dB (SD = 11.7). Of these, 49.3 percent (n = 687) were boys (mean: 34.1 [SD = 11.8]) and 50.7 percent (n = 706) were girls (mean: 33.5 [SD = 11.7]). Characteristics of the study population in total by three categories of traffic noise exposure are shown in Table 1. Characteristics by gender and noise categories are shown in Table S1 (supplementary material). Household income and maternal education tended to be lower for children exposed to higher noise levels. Highly exposed children were also more often of non-Western ethnicity, lived in apartments and closer to the city center, and were more often exposed to rail traffic noise.

### 3.2. Study Sample versus Non-Participants 

Comparing participants with all the non-participants, the two groups were equal in terms of gender and ethnicity. However, the study sample had mothers with higher education (*p* < 0.001), higher gross household income (1,009,347 vs. 919,559 NOK, *p* < 0.0001), fewer children with siblings under 4 years of age (*p* < 0.000), fewer children living in detached houses, and more living in apartment buildings (*p* < 0.000). However, none of these variables changed the odds ratio between road traffic noise and sleep. Comparing participants with non-participants living in Oslo at age 7, participants had slightly lower mean road traffic noise exposure than non-participants (L_en_ 47.2 dB vs. 48.1 dB, *p* = 0.0001).

### 3.3. Main Results

**Figure 2:**
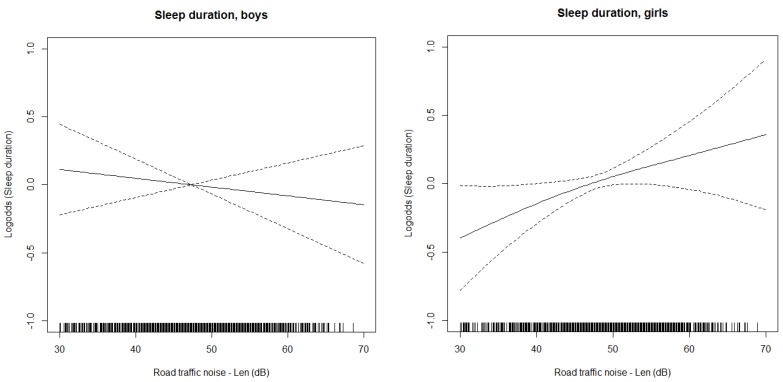
Splines with 95 % confidence limits of the associations between night-time road traffic noise from 30 dB and sleep duration for boys and girls separately. The vertical lines on the x-axis indicate number of observations. General Additive Model adjusted for age, household income, urbanity, maternal education, ethnicity, season, younger siblings, residential type of building (dB = decibel).

A trend, but no statistically significant associations were found between road traffic noise and sleep duration in the fully adjusted model (Table 2). We found a statistically significant interaction between road traffic noise and gender (*p* = 0.06). When stratifying by gender, an association between road traffic noise and sleep duration was found for girls, with an OR in the full model, similar to the OR in the crude model and in the model with the minimal adjustment set. For boys, no association was found with either of the models (see Table 2). The splines of the association between noise and sleep duration shown in Figure 2a and b show an exposure-response relationship for girls, but not for boys. The results for girls indicate that, per 10 decibel increase in road traffic noise level exposure, the odds for sleeping 10 h or less, as compared to more than 10 h, increased by 21 percent. Similarly, the odds for sleeping less than 10 h, as compared to 10 h or more, increased with 21 percent. 

There were no statistically significant interactions between road traffic noise and household income (*p* = 0.57), or between road traffic noise and maternal education (*p* = 0.37). The ORs remained similar or slightly lowered when children not living with both parents were excluded, both in the full model (OR = 1.03, 95% CI: [0.92, 1.15]) for girls (OR = 1.21, 95% CI: [1.03, 1.42]) and for boys (OR = 0.87, 95% CI: [0.74, 1.02]). Including rail traffic noise as a covariate did not change the estimates for road traffic noise (see Table 2). The mean level of rail traffic noise exposure did not differ between genders (F = 0.05, *p* = 0.83), nor did the proportion of rail noise exposed children (*X^2^*(1) = 0.52, *p* = 0.47).

Only 76 (2.9%) of the children in the study sample were reported as having sleep problems at age 7: 38 boys and 38 girls. More than half of the children with sleep problems (52.6%) also had sleep duration less than 10 h, whereas only 13.2% of children with sleep problems had sleep duration of more than 10 h. No statistically significant association between road traffic noise and sleep problems were found in the logistic regression models (OR = 1.09, 95% CI = [0.80, 1.50]). The interaction between road traffic noise and gender was statistically significant (*p* = 0.09). The estimates in the stratified model were for boys: OR = 0.87, 95% CI = [0.56, 1.36]; and for girls: OR = 1.36, 95% CI = [0.85, 2.16]).

## 4. Discussion

### 4.1. Summary of Findings and Comparison with Existing Literature 

The present study investigated the association between nocturnal road traffic noise exposure and sleep duration and sleep problems in children aged 7 years. These associations were not statistically significant, although the effect estimates were in the expected direction. Effect modification by gender was found, with a statistically significant association between road traffic noise and sleep duration for girls, but not boys. The estimates in the gender stratified analysis with sleep problems as outcome resembled those found for sleep duration, although the sleep duration analyses are likely a bit underpowered with wide confidence intervals. Conclusions should therefore not be made based on sleep problems analyses alone. Still, the results for the two different sleep outcomes seem to be mutually supportive, and add strength to the findings.

In sum, our hypothesis was partly strengthened with the association found for girls. In the full sample, a trend was detected, which might have been statistically significant with a larger study sample, but this trend was due to the association among the girls. 

Previous studies on traffic noise and sleep in children have found associations for some sleep outcomes [13,24], but the evidence for children is very limited [14]. None of the studies on children examined the association with sleep duration, with the exception of the sub-study of 80 children by Öhrström, Hadzibajramovic, Holmes, and Svensson [13]. In that study, sleep duration was measured with actigraphs, and no association was found. That finding matches our finding for the total sample. Tiesler et al. [24] found no associations between road traffic noise at the most exposed façade and any sleeping problems, as in our study, although they found associations when noise at the least exposed façade was used.

Concerning the gender differences found, no study to date has investigated gender differences in the association between traffic noise and sleep among children. The few existing studies on gender differences in adults have yielded mixed results [30,31,32]. Previous studies with parent reported sleep (not including noise) have found gender differences, with longer sleep duration for girls [33,50], although this finding is not entirely consistent [22]. The results from the present study also suggest a tendency for girls to sleep longer than boys. However, it is not clear whether and to what extent gender differences in sleep can explain the gender differences in the association between noise and sleep found in the present study. Rail traffic noise exposure did not differ between genders, making additional disturbance from that noise source an unlikely explanation for the gender differences. Girls might be more sensitive to noise than boys. Unfortunately, we had no information to investigate this further.

As is the case for gender differences, the literature on SES’s role in the association between noise and sleep is very scarce. Typically, studies have only assessed SES and sleep or SES and noise. Poorer sleep environment and pre-sleep worries have been proposed as some of the factors mediating the association between SES and sleep [27]. If low-SES children are more vulnerable when it comes to sleep, then it is likely that their sleep would be disrupted at lower road traffic noise levels, compared to high-SES children. Perhaps a little unexpected, neither household income, nor mothers’ education modified the association between road traffic noise and sleep duration. The mean household income and the education level in the present study sample was higher than for the MoBa participants that were not included in our study sample. Thus, the socioeconomic status of our study population was relatively high. It might be that the SES level was too high in the present study to detect a potential moderating effect of SES, and that exposure-response curves would have been different in more vulnerable SES groups.

The sensitivity analysis including only children living with both parents made almost no change to the estimates. This is not surprising, since the number of children not living with both parents was low (11 percent, n = 294). The majority of the children were not exposed to rail traffic noise, or the noise levels were very low. In sum, it is not surprising that rail traffic noise had little impact on the road traffic noise estimates. 

It has been previously reported that children are less easily awakened by noise compared to adults [26] and therefore seem to be more protected against sleep disruptions due to noise than adults. However, children may cope with noise in a different, less adequate way [51]. Children also need more sleep [50] and usually go to bed early in the evening, when traffic noise exposure is higher than during the night [15]. Even though sleep duration or awakenings are not severely affected by noise, other reactions during sleep may occur. Cardiac responses to noise, such as elevated heart rate, can take place even in the absence of awakenings [11]. It is also evident from research on adults that traffic noise can disturb sleep without necessarily affecting sleep duration or awakenings, but by causing changes in sleep stages [8]. Habituation to traffic noise is found to occur for some outcomes, such as cortical arousals, whereas other outcomes, such as cardiac responses, habituate to a much lesser degree [11,52]. Based on the above, one cannot conclude that boys are not disturbed by road traffic noise during sleep, despite the absence of a significant association in the present study. Other sleep disruptions due to noise might have occurred that were not detected by the parental reported sleep duration. 

### 4.2. Strengths and Limitations

The present study has several strengths. First, this study is the first population based study on traffic noise and sleep to include children as young as 7 years. Second, the number of participants is fairly high, reducing the risk of reporting false negatives (Type II error). Third, covariates were selected with the aid of the DAG framework, reducing the chances of obtaining a biased result. Much of the covariates were obtained from the Medical Birth Registry of Norway and Statistics Norway, ensuring that the number of missing values were kept to a minimum level, and increasing the likelihood that the values were correct. Fourth, the study sample had a large contrast in noise exposure, strengthening the opportunity to detect associations. Further, several factors were controlled for in the full model or addressed in sensitivity analyses that likely have an impact on the total noise that children are exposed to in their homes. This included rail traffic noise (train, tram, and subway) and urbanity (proximity to the Oslo city center). Urbanity likely reflects exposure to noise from other sources than traffic. For example, people living in the most central areas of the city have a higher chance of being exposed to noise from bars, pubs, people talking or shouting in the streets, sirens, etc. Also, accounting for the fact that some children likely switched between addresses, since they had parents who lived apart, increased the precision of the exposure. Finally, and importantly, using two different kinds of sleep outcomes that both pointed in the same direction, strengthens the findings.

The study also has some limitations that need to be addressed. Only subjective reports by mothers were used as a measure of children’s sleep. Objective sleep measures, taking into account sleep efficiency, could have given some insight into this, and would probably have been a more sensitive measure of sleep than reported sleep duration. However, even though self-reported sleep is inferior to objective measures of sleep, good correspondence between objective measures of sleep (as measured by actigraphy) and parental perception of sleep latency and sleep duration are reported [53]. The tendency was that the shorter the sleep duration, the higher the proportion of children with reported sleep problems. Although sleep duration does not necessarily reflect sleep problems, but may rather be related to lifestyle factors, shorter sleep duration could reflect problems falling asleep due to traffic noise, or premature awakening in the morning. Self-reported short sleep duration is consistently found to be associated with negative health outcomes [17]. Therefore, sleep duration seems to a certain extent to reflect sleep problems, and is an informative sleep parameter when it comes to assessing risks for health or daily functioning impairments. In addition, the majority of children in the present study slept approximately 10 hours per night, which is close to the average sleep duration for 7 to 8 year-olds found by others [22]. Unfortunately, no information about bedroom location (towards most or least exposed facade) was available. However, as previously pointed out, when the noise exposure level outside the most exposed façade is used, the result is likely an underestimated noise-sleep effect for those with a bedroom facing the most exposed façade, since some of the study participants have their bedroom facing the least exposed façade [47]. According to this reasoning, including information on bedroom side would probably have strengthened the association between road traffic noise and sleep duration in the present study. Furthermore, modelled levels of outdoor road traffic levels and indoor noise levels (from road traffic) are not necessarily highly correlated [12,54]. How well road traffic noise modelled outdoors reflect the indoor noise level depends on a number of factors including, façade and window insulation, type of ventilation, and whether windows are kept open or closed, factors which were not available in our study. Thus, the possibility of misclassification due to underestimation or overestimation of true individual noise exposure cannot be excluded.

As demonstrated from the analyses of the study sample versus non-participants, the study sample had higher socioeconomic status than the non-participants, as evidenced by higher income and education. The study sample also had lower average road traffic noise exposure, but although significant, the difference was small (less than 1 dB). The covariates that differed significantly between the two groups made almost no change in the odds ratio between road traffic noise and sleep duration.

## 5. Conclusions

Increased nocturnal road traffic noise was associated with reduced sleep duration for girls, but not boys. The scientific knowledge on the association between noise and sleep in children is still scarce. Future studies on road traffic noise and children’s sleep should be longitudinal, investigate further possible gender differences, and also explore differences in SES, with a wider range of SES levels, to see whether low-SES children are more vulnerable to road traffic noise when it comes to sleep. Both large epidemiological studies and experimental studies should be conducted, including studies with objective measures and measures of after-effects (i.e., sleepiness, cognitive functioning).

## Figures and Tables

**Table 1:** Characteristics of the study population in total, by three categories of traffic noise exposure (L_en_).

Covariate	Total Study Sample (n = 2665)			
	**<40 dB**	**40–50 dB**	**>50 dB**	**Total**
n (%)	455 (17.1)	1321(49.6)	889 (33.4)	2665
% male	54.3	50.3	47.6	50.1
Sleep duration (%)				
>10 h	19.0	49.0	32.0	27.5
10 h	16.6	50.3	33.1	57.5
<10 h	15.5	47.8	36.8	15.0
Sleep problems (% yes)	2.6	2.4	3.6	2.9
Gross annual household income ^a^ (NOK), mean (SD)	1,057,762 (686,380)	1,070,305 (923,924)	893,990 (598,621)	100,9347 (793,343)
Season, questionnaire completion (%)				
Winter	19.5	46.8	33.7	23.6
Spring	16.2	48.5	35.3	23.9
Summer	15.6	51.9	32.6	20.8
Fall	16.9	50.9	32.2	31.7
Urbanity ^b^ (%)				
Outskirts	20.2	53.3	26.5	71.8
Semi-central	11.1	48.6	40.2	20.5
Center	3.4	17.1	79.5	7.7
Age (months), mean (SD)	85.5 (1.5)	85.4 (1.6)	85.4 (1.6)	85.4 (1.6)
Mother’s education (%)				
>4 years university/college	17.2	52.1	30.7	41.6
≤4 years university/college	17.8	48.4	33.7	44.2
High school	14.2	45.8	40.0	14.3
Ethnicity ^c^ (%)				
Non-Western	15.1	39.6	45.3	10.4
Western	17.3	50.7	32.0	89.6
Siblings < age 4 (%)				
Yes	18.1	49.8	32.2	37.0
No	16.5	49.4	34.1	63.0
Type of building (%)				
Detached house	19.5	57.7	22.8	22.4
Semi-detached	19.4	54.0	26.6	46.3
Apartment	12.0	37.2	50.8	31.3
Rail traffic noise (L_en_)				
0 dB	59.6	48.4	40.7	47.7
≤30 dB	20.2	24.8	17.3	21.5
>30 dB	20.2	26.9	42.0	30.8

^a^ Adjusted according to consumer price index. ^b^ Outskirts: outside the Ring 3 road. Between: between roads Ring 2 and Ring 3; Center: inside Ring 2 road. The covariate indicated how far the children lived from the city center. ^c^ Dichotomized according to Statistics Norway [49]. NOK = Norwegian kroner. SD = standard deviation.

**Table 2:** Odds ratios (per 10 dB increase in noise) for associations between road traffic noise (L_en_) and sleep duration, with 95% confidence intervals.

Analysis	Total	Girls	Boys
Crude ^a^ (n = 2665)	1.08 (0.98, 1.19)	1.19 (1.03, 1.37)	0.99, (0.86, 1.14)
Min. adj. set ^b^ (n = 2665)	1.07 (0.96, 1.18)	1.17 (1.01, 1.36)	0.98 (0.85, 1.13)
Full ^c^ (n = 2665)	1.05 (0.94, 1.17)	1.21 (1.04, 1.41)	0.91 (0.78, 1.06)
Live with both parents (n = 2371)	1.03 (0.92, 1.15)	1.21 (1.03, 1.42)	0.87 (0.74, 1.02)
Full with rail traffic noise ^d^ (n = 2665)	1.04 (0.94, 1.16)	1.18 (1.01, 1.38)	0.93 (0.80, 1.08)

^a^ adjusted for age and gender in the total sample; adjusted for age in the stratified analyses. ^b^ additionally adjusted for income, season, and urbanity. ^c^ additionally adjusted for mother’s education, ethnicity, siblings in child’s household, and type of residential building. ^d^ Rail traffic noise added as a covariate in the full model, categorized as 0 dB, >0 to ≤30 dB, >30 dB.

## Supplementary Materials

The following are available online at www.mdpi.com/1660-4601/14/5/491/s1, Figure S1: Directed acyclic graphs used in the selection of covariates; Table S1: Characteristics of the study sample, by gender and noise categories.
