# Supplementary material for: Nocturnal Road Traffic Noise Exposure and Children’s Sleep Duration and Sleep Problems
Source: Int J Environ Res Public Health. 2017 May 6;14(5):491. doi: 10.3390/ijerph14050491 (PMC5451942; doi:10.3390/ijerph14050491)
Supplement: Supplementary file 1 [file ijerph-14-00491-s001.pdf]

Supplemental Material

Nocturnal Road Traffic Noise Exposure and Children's Sleep Duration and Sleep Problems

Weyde, Kjell Vegard[a]
Krog, Norun Hjertager[a]
Oftedal, Bente[a]
Evandt, Jorunn[a]
Magnus, Per[b, c]
Øverland, Simon[d, e]
Clark, Charlotte[f]
Stansfeld, Stephen[f]
Aasvang, Gunn Marit[a]

[a]Department of Air Pollution and Noise, Norwegian Institute of Public Health, Oslo, Norway
[b]Domain of Health Data and Digitalization, Norwegian Institute of Public Health, Oslo, Norway
[c]Institute of Health and Society, Faculty of Medicine, University of Oslo, Oslo, Norway
[d]Division of Mental Health, Norwegian Institute of Public Health, Bergen, Norway
[e]Faculty of Psychology, University of Bergen, Bergen, Norway
[f]Centre for Psychiatry, Wolfson Institute of Preventive Medicine, Barts and the London School of Medicine, Queen Mary University of London, London, United Kingdom

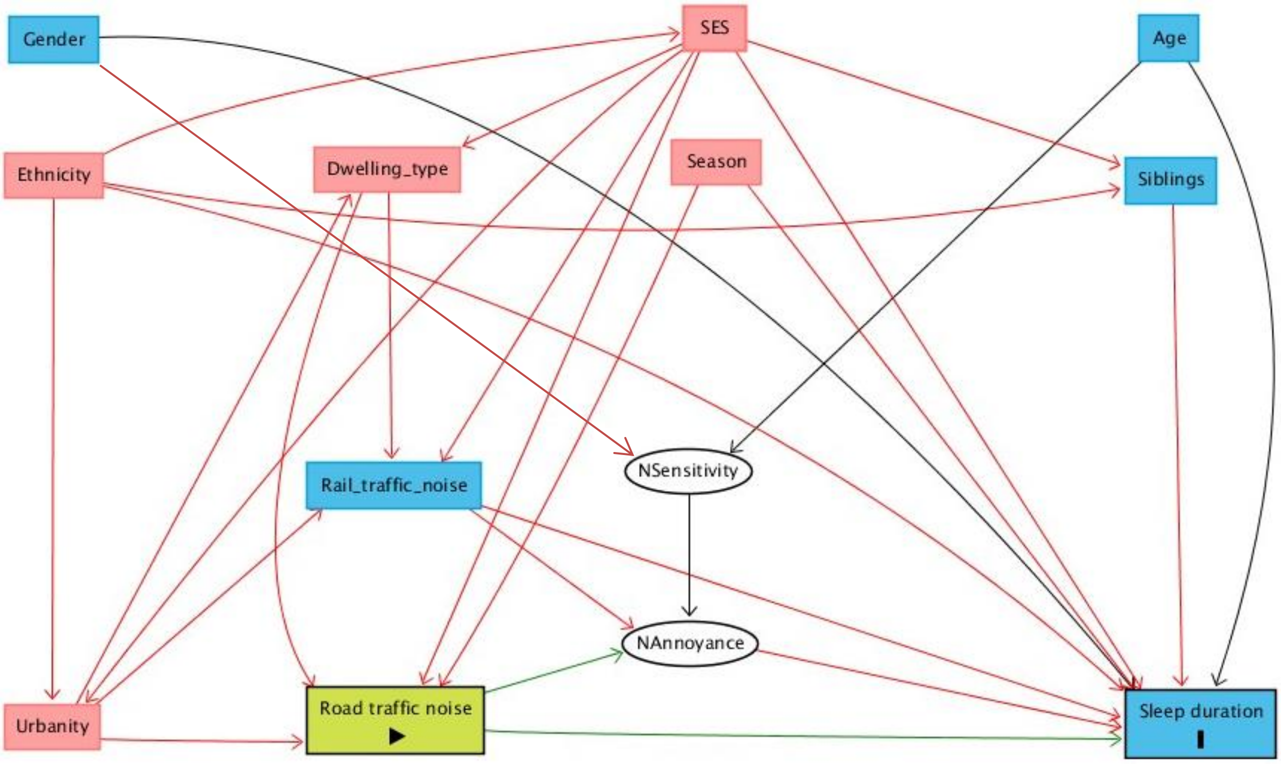

Figure S1: Directed acyclic graphs used in the selection of covariates.
SES includes annual gross household income and maternal education

Table S1: Characteristics of the study sample, by gender and noise categories.

	Girls (N=1331)				Boys (N=1334)			
	<40dB	40-50dB	>50dB	Total	<40dB	40-50dB	>50dB	Total
N (%)	208 (15.6)	657 (49.4)	466 (35.0)	1331	247 (18.5)	664 (49.8)	423 (31.7)	1334
Sleep duration (%)								
>10h	19.2	50.0	30.8	N=386 (29.0%)	18.8	48.0	33.2	N=346 (25.9%)
10h	14.3	49.5	36.2	N=768 (57.7%)	18.8	51.1	30.1	N=765 (57.4%)
<10h	13.6	47.5	39.0	N=177 (13.3%)	17.0	48.0	35.0	N=223 (16.7%)
Sleep problems (% yes)	1.9	2.3	4.1	2.9	3.2	2.6	3.1	2.9
Gross annual household income[a] (NOK), mean (SD)	1056719 (800632)	1038648 (649425)	943156 (749891)	1008039 (711831)	1058640 (574518)	1101628 (1131490)	839826 (358902)	1010653 (867338)
Season, questionnaire completion (%)								
Winter	17.7	46.6	35.7	22.1	21.1	47.0	31.9	25.2
Spring	13.8	49.2	37.1	22.9	18.4	47.9	33.7	24.9
Summer	16.0	49.5	34.6	22.6	15.1	54.8	30.2	18.9
Fall	15.3	51.3	33.4	32.4	18.6	50.5	30.9	31.0
Urbanity[b] (%)								
Outskirts	18.3	53.3	28.5	71.2	22.2	53.4	24.5	72.3
Semi-central	11.4	47.9	40.8	21.2	10.9	49.4	39.6	19.9
Center	3.0	16.8	80.2	7.6	3.9	17.3	78.9	7.8
Age (months), mean (SD)	85.5 (1.5)	85.4 (1.6)	85.4 (1.6)	85.4 (1.6)	85.4 (1.5)	85.4 (1.5)	85.4 (1.7)	85.3 (1.6)
Mother's education (%)								
>4 years univ/college	15.3	52.2	32.5	41.2	19.1	52.0	28.9	42.0
≤4 years univ/college	16.5	47.5	36.0	44.1	19.2	49.3	31.5	44.2
High school	13.8	46.9	39.3	14.7	14.7	44.6	40.8	13.8
Ethnicity[c] (%)								
Non-western	12.7	40.5	46.8	9.5	17.1	38.8	44.1	11.4
Western	15.9	50.3	33.8	90.5	18.7	51.2	30.1	88.6
Siblings < age 4 (%)								
Yes	16.7	50.6	32.7	37.4	19.5	49.0	31.6	36.6
No	15.0	48.8	36.4	62.6	18.0	50.2	31.8	63.4
Type of building (%)								
Detached house	14.7	57.5	27.8	20.0	23.3	57.9	18.8	24.7
Semi-detached	17.8	54.8	27.5	48.7	21.1	53.2	25.7	44.0
Apartment	13.0	35.7	51.3	31.3	11.0	38.6	50.4	31.3
Rail traffic noise (L_{en})								
0 dB	61.5	46.3	41.4	47.0	57.9	50.5	40.0	48.5
≤30 dB	22.6	26.2	17.4	22.5	18.2	23.3	17.3	20.5
>30 dB	15.9	27.6	41.2	30.5	23.9	26.2	42.8	31.0

[a]Adjusted according to consumer price index

[b]Outskirts: outside the Ring 3 road; Between: between roads Ring 2 and Ring 3; Center: inside Ring 2 road. The covariate indicated how far the children lived from the city center.

[c]Dichotomized according to Statistics Norway [50].
